# Environmental factors associated with juvenile idiopathic inflammatory myopathy clinical and serologic phenotypes

**DOI:** 10.1186/s12969-022-00684-9

**Published:** 2022-04-12

**Authors:** Jonathan C. Scalabrini, Adam I. Schiffenbauer, Payam Noroozi Farhadi, Rita Volochayev, Nastaran Bayat, Anna Jansen, Ira N. Targoff, Frederick W. Miller, Lisa G. Rider

**Affiliations:** 1 Environmental Autoimmunity Group, Clinical Research Branch, National Institute of Environmental Health Sciences, National Institutes of Health, Clinical Research Center Room 4-2352, 10 Center Drive, MSC 1301, MD 20892-1301 Bethesda, USA; 2 Veteran’s Affairs Medical Center, University of Oklahoma Health Sciences Center, and Oklahoma Medical Research Foundation, Oklahoma City, OK USA

**Keywords:** Juvenile myositis, Environmental factors, Phenotype, Myositis autoantibodies

## Abstract

**Background:**

Environmental exposures have been associated with the juvenile idiopathic inflammatory myopathies (JIIM). We undertook a questionnaire-based study to evaluate patient-reported exposures as possible risk factors for JIIM.

**Findings:**

One-hundred-seven patients with JIIM were enrolled in a myositis natural history protocol and completed environmental questionnaires. Frequencies of exposures in clinical and myositis-specific autoantibody (MSA) groups were examined. Patients with juvenile dermatomyositis (JDM) and juvenile connective tissue myositis (JCTM) more frequently received an immunization within 1 year of diagnosis compared to juvenile polymyositis (57.5 and 71.4% vs 0.0%, *p* ≤ 0.017). JCTM patients were more often underweight at diagnosis relative to JDM patients (42.9% vs 7.0%, *p* = 0.002). MSA-negative patients more frequently had gastroenteritis within a year of diagnosis compared to patients with anti-MDA5 autoantibodies (28.6% vs 0.0%, *p* = 0.032). Heavy exercise was more frequent in MSA-negative and anti-MDA5 groups compared to the anti-TIF-1 autoantibody group (42.9 and 35.3% vs. 9.0%, *p* ≤ 0.047). Medications received within 1 year of diagnosis were more frequent in MSA-negative patients relative to those with anti-MDA5 autoantibodies (92.9% vs. 52.8% *p* = 0.045). Being breastfed > 6 months was more frequent in MSA-negative patients (88.9%) compared to anti-TIF-1 and anti-MDA5 autoantibody groups (41.2 and 28.6%, *p* ≤ 0.036).

**Conclusions:**

Certain environmental exposures prior to diagnosis differed among clinical and serologic subgroups of JIIM, suggesting additional exposures to be explored as possible risk factors for JIIM phenotypes.

**Supplementary Information:**

The online version contains supplementary material available at 10.1186/s12969-022-00684-9.

## Background

The juvenile idiopathic inflammatory myopathies (JIIM) are a heterogenous group of rare systemic autoimmune diseases characterized by chronic muscle inflammation and proximal weakness [1]. These diseases have been divided into more homogenous clinicopathologic and autoantibody subgroups that share clinical features, outcomes, and immunogenetic risk factors [2–4].

Although the causes of these disorders remain unknown, evidence has suggested that these conditions result from the interaction of environmental exposures and genetic risk factors [5]. A case-control study of newly diagnosed juvenile dermatomyositis (JDM) patients found a higher frequency of antecedent infections relative to children with juvenile idiopathic arthritis (JIA) and healthy children, while in a JDM inception cohort and two nationwide registry studies, a high prevalence of respiratory illnesses prior to disease onset was observed [6–9]. Among non-infectious exposures, residential ultraviolet radiation (UVR) within 30 days prior to diagnosis has been associated with certain subgroups, including JDM and anti-TIF-1 autoantibodies [10]. In a case-control study from Brazil, prenatal exposure to air pollution, maternal chalk dust occupation, and maternal tobacco smoke were identified as risk factors for JDM [11].

Given the existence of multiple JIIM subtypes with possibly differing etiologies, we undertook this questionnaire-based study to examine whether environmental exposures temporally associated with the diagnosis of JIIM vary among clinical or serologic phenotypes. Compared to a prior nationwide study, this study involved a more extensive environmental questionnaire, probed a larger number of exposures over a longer period prior to diagnosis, and examined major serologic subgroups not previously examined [7].

## Findings

### Methods

One hundred seven patients with probable or definite JIIM as per Bohan and Peter criteria were enrolled in the National Institute of Environmental Health Sciences (NIEHS) myositis natural history protocol between 2009 and 2020 [12]. The population represents a referral cohort, with patients residing in 17 states and all 9 United States (U.S.) climate regions. Participants completed the myositis Brief Environmental Questionnaire (BEQ) and other study questionnaires, provided a blood sample for myositis autoantibody testing by immunoprecipitation and immunoprecipitation-immunoblotting [13], and were evaluated by a rheumatologist.

Patients completed the BEQ, which focuses on exposures within 12 months of diagnosis, but includes some exposures up to 5 years prior to diagnosis (Supplementary Table). The BEQ was developed from Swedish Epidemiological Investigation of RA (EIRA) study exposure questionnaire, as well as questionnaires from NIEHS environmental exposure studies of myositis and other autoimmune diseases and others, with the intention of capturing a broad range of environmental exposures in children and adults [14–16]. Both the primary care records for the year prior to diagnosis and initial diagnostic records of the treating pediatric rheumatologist were reviewed to verify some of the queried exposures, particularly infections, medications and vaccines. All study questionnaires were reviewed with the parent and patient in the clinic following completion to review any inconsistencies or missing data. Major psychosocial stress scores were computed by summing the impact score of eight major stressors, with a score for each ranging from − 2, indicating a very negative impact, to + 2, indicating a very positive impact. In addition, 73 of 107 participants had pregnancy and prenatal histories obtained by a prenatal and pollution exposure questionnaire [11] or by a pregnancy questionnaire in the NIEHS Study of Twins/Siblings Discordant for Systemic Rheumatic Diseases [17]; these questionnaires were identical.

Statistical analysis was performed using GraphPad Prism version 9.1.2 (GraphPad Software, San Diego, CA) and included Chi-squared analysis (or Fisher’s exact test when appropriate) by clinical or autoantibody subgroup to determine differences in proportions of patients with given exposures. The Kruskal-Wallis test was used to compare median values among subgroups. Odds ratios (OR) and their associated 95% confidence intervals (CI) were also computed. A *p* value of less than 0.050 was considered significant in this exploratory study.

### Results

Table 1 provides the demographic features of the major clinical and serologic subgroups. The majority of patients in each subgroup were female, other than patients negative for myositis-specific autoantibodies (MSA-negative). JDM patients were younger at diagnosis compared to juvenile connective tissue myositis (JCTM) and juvenile polymyositis (JPM) patients, while MSA-negative patients were older at diagnosis compared to the other serologic subgroups. Within the clinical and autoantibody subgroups, disease duration was comparable, with a majority of patients enrolled within 3 years of diagnosis. Furthermore, the delay between symptom onset and diagnosis, defined as delay to diagnosis, did not differ between clinical and serologic subgroups and was less than 1 year for the majority of patients. Patients were primarily Caucasian, with the largest percentage in the anti-TIF-1 autoantibody group. A larger proportion of anti-MDA5 autoantibody positive patients were Black or other races. Most parents had a college or graduate level degree, except in the JPM and anti-TIF-1 autoantibody positive groups. A greater proportion of anti-MDA5 autoantibody positive patients lived in large urban areas compared to MSA-negative patients, and median household income was similar among clinical and serologic subgroups.

**Table 1:** Demographic features of the JIIM participants by clinical and serologic subgroup included in the environmental analysis

	Clinical Subgroups^1^			Serologic Subgroups^2^			
	JDM *n* = 87 N (%) or Median [IQR]	JCTM^3^*n* = 14 N (%) or Median [IQR]	JPM *n* = 5 N (%) or Median [IQR]	Anti-TIF-1 (p155/140) *n* = 33 N (%) or Median [IQR]	Anti-NXP2 (MJ) *n* = 30 N (%) or Median [IQR]	Anti-MDA5 *n* = 17 N (%) or Median [IQR]	MSA Negative *n* = 14 N (%) or Median [IQR]
**Sex**							
Female	54 (62.1)	9 (64.3)	3 (60.0)	26 (78.8)^a^	20 (66.7)	9 (52.9)	5 (35.7)
**Age at Diagnosis (Years)**							
Median	6.7 [4.0–10.3]^e^	10.9 [7.3–13.8]	12.2 [6.9–14.7]	6.1 [2.8–10.0]	7.4 [5.4–12.4]	6.8 [4.9–9.7]	11.8 [5.3–14.6]^f^
**Disease Duration (Years)**							
Median	1.5 [0.5–3.5]	1.6 [0.6–8.4]	1.5 [0.7–3.1]	2.7 [0.7–3.9]	2.0 [0.8–4.7]	1.0 [0.4–1.9]	1.0 [0.6–3.1]
**Diagnosis Delay (Years)**							
Median	0.4 [0.0–1.0]	0.3 [0.0–1.5]	0.0 [0.0–4.1]	0.2 [0.0–1.0]	0.9 [0.3–2.5]	0.3 [0.0–0.7]	0.4 [0.1–1.7]
**Race**							
Caucasian	61 (70.1)	6 (42.9)	3 (60.0)	28 (84.8)^b^	18 (60.0)	6 (35.3)	9 (64.3)
Black	6 (6.9)	3 (21.4)	1 (20.0)	1 (3.0)	3 (10.0)	4 (23.5)^c^	1 (7.1)
Hispanic	13 (14.9)	2 (14.3)	1 (20.0)	4 (12.1)	6 (20.0)	2 (11.8)	2 (14.3)
Other	7 (8.0)	3 (21.4)	0 (0.0)	0 (0.0)	3 (10.0)	5 (29.4)^d^	2 (14.3)
**Parental Education**							
College or Graduate Degree	51 (60.0)	7 (53.8)	1 (25.0)	14 (45.2)	20 (65.5)	9 (52.9)	10 (71.4)
**Urban residential location**^4^							
Metropolitan area ≥ 1 million residents	51 (63.0)	7 (53.8)	3 (60.0)	19 (59.4)	18 (62.1)	13 (86.7)^g^	6 (46.2)
Metropolitan area with less than 1 million residents	18 (22.2)	3 (23.1)	2 (40.0)	9 (28.1)^h^	5 (17.2)	0 (0.0)	5 (38.5)^i^
Non-metropolitan area	12 (14.8)	3 (23.1)	0 (0.0)	4 (12.5)	7 (24.1)	2 (13.3)	2 (15.4)
**Household Income**^5^							
Median	$33,663 [$13,967–$75,057]	$64,156 [$15,591–$89,675]	$55,378 [$26,293–$76,561]	$68,371 [$45,459–$92,141]	$59,630 [$49,599–$86,282]	$61,066 [$40,982–$99,875]	$55,378 [$11,961–$79,220]

*Abbreviations*: *JDM* juvenile dermatomyositis, *JPM* juvenile polymyositis, *JCTM* juvenile connective tissue myositis, *IQR* interquartile range, *MSA* myositis- specific autoantibody

For diagnosis delay, three patients are missing, including two JDM and one JCTM from the clinical subgroups, and one autoantibody negative patient from the serologic subgroups. For parental education level, four patients are missing, including two JDM, one JCTM, and one JPM patients from the clinical subgroups, and two anti-TIF-1 and one anti-NXP2 autoantibody positive patients from the serologic subgroups. For household income level, six patients are missing, including five JDM and one JCTM from the clinical subgroups, and one anti-TIF-1, one anti-NXP2, two anti-MDA5 autoantibody patients, and one autoantibody negative patient from the serologic subgroups

^1^One patient with immune-mediated necrotizing myopathy was excluded from all analyses

^2^Patients excluded from the serologic subgroup analysis were two patients with anti-signal recognition particle, one with anti-Mi2 autoantibodies, two with indeterminate myositis autoantibodies, and three that had no myositis autoantibody results available. Three patients with anti-Jo1 autoantibodies and one with anti-PL-12 autoantibodies (i.e., those with anti-synthetase autoantibodies) were examined only descriptively and not included in Table 2

^3^Those with JCTM, met the criteria for myositis and at least one other autoimmune disease. The overlapping autoimmune diseases were systemic lupus erythematosus (four patients), celiac disease (three patients), scleroderma (two patients), and linear scleroderma, autoimmune hepatitis, type 1 diabetes mellitus, alopecia areata, and juvenile idiopathic arthritis (one patient each)

^4^Based on Urban Influence Codes of U.S. Department of Agriculture, using residential zip code at diagnosis and U.S.census data

^5^ From the American Community Survey, geocoding zip code at diagnosis to the centroid of the census tract level

^a^*P* = 0.007 between anti-TIF-1 autoantibody positive and MSA-negative; ^b^*P* = 0.001 between anti-TIF-1 and anti-MDA5 autoantibody positive; ^c^*P* = 0.040 between anti-MDA5 and anti-TIF-1 autoantibody positive; ^d^*P* = 0.030 between anti-MDA5 and anti-TIF-1 autoantibody positive; ^e^*P* = 0.034 between JDM and JCTM; ^f^*P* = 0.036 between anti-TIF-1 autoantibody positive and MSA-negative; ^g^*P* = 0.042 between anti-MDA5 autoantibody positive and MSA-negative; ^h^*P* = 0.042 between anti-TIF-1 autoantibody positive and anti-MDA5 autoantibody positive; ^i^*P* = 0.013 between MSA-negative and anti-MDA5 autoantibody positive

No differences were observed among clinical subgroups in the frequency of any infections within 12 months of diagnosis, or by specific infection type, including influenza, Group A streptococcal pharyngitis, upper respiratory infection, urinary tract infection, gastroenteritis, pneumonia, and hepatitis (Table 2). Gastroenteritis was more frequent among MSA-negative patients relative to anti-MDA5 autoantibody positive patients within a year of diagnosis (28.6% vs. 0.0%, *p* = 0.032).

**Table 2:** Environmental exposures documented in selected juvenile idiopathic inflammatory myopathy phenotypes

	Clinical Subgroup					Myositis-Specific Autoantibody Subgroup					
Environmental Exposure	JDM N (%) or Median [IQR] *n* = 87	JCTM N (%) or Median [IQR] *n* = 14	JPM N (%) or Median [IQR] *n* = 5	*P* Value	OR (95% CI)	Anti-TIF-1 N (%) or Median [IQR] *n* = 33	Anti-NXP2 N (%) or Median [IQR] *n* = 30	Anti-MDA5 N (%) or Median [IQR] *n* = 17	MSA Negative N (%) or Median [IQR] *n* = 14	*P* Value	OR (95% CI)
**Exposures within 1 Year of Diagnosis**											
**Infections**											
Any Infection	49 (56.3)	5 (35.7)	4 (80.0)	0.294		19 (57.6)	18 (60.0)	6 (35.3)	9 (64.3)	0.135	
Gastroenteritis	9 (10.3)	1 (7.1)	1 (20.0)	0.468		4 (12.1)	3 (10.0)	0 (0.0)^d^	4 (28.6)^d^	0.032^d^	NC^d^
Strep Throat	20 (23.2)	2 (14.3)	1 (20.0)	0.729		4 (12.1)	9 (31.0)	3 (17.6)	5 (35.7)	0.102	
URI	13 (14.9)	1 (7.1)	0 (0.0)	0.685		5 (15.2)	5 (16.7)	2 (11.8)	1 (7.1)	0.647	
Influenza	7 (8.0)	0 (0.0)	0 (0.0)	0.589		4 (12.1)	1 (3.3)	1 (5.9)	1 (7.1)	0.357	
**UV Exposure**											
Any Sunburn	43 (50.0)	8 (57.1)	3 (60.0)	0.775		16 (48.5)	14 (46.7)	6 (37.5)	8 (57.1)	0.549	
Median Number of Sunburns	0.5 [0.0–2.0]	1.0 [0.0–1.5]	1.0 [0.0–1.0]	0.823		0.0 [0.0–20.0]	0.0 [0.0–2.0]	0.0 [0.0–1.0]	1.0 [0.0–1.3]	0.786	
**Heavy Exercises**											
Any Heavy Exercise	29 (33.3)	6 (42.9)	2 (40.0)	0.550		9 (27.3)	8 (26.7)	8 (47.0)	8 (57.1)	0.091	
Exercise Resulting in Muscle Pain	18 (20.7)	4 (28.6)	1 (20.0)	0.498		3 (9.0)^e^	6 (20.0)	5 (29.4)	6 (42.9)^e^	0.013^e^	7.5^e^ (1.6–30.5)
Prolonged Running	15 (17.2)	2 (14.3)	0 (0.0)	0.579		3 (9.0)^f^	4 (13.3)	6 (35.3)^f^	2 (14.3)	0.047^f^	5.5^f^ (1.3–21.8)
Heavy Sports Activity	24 (27.6)	6 (42.9)	1 (20.0)	0.344		8 (24.2)	6 (20.0)	8 (47.0)	7 (50.0)	0.074	
**Medications**											
Any Medication	59 (67.8)	10 (71.4)	5 (100)	0.316		21 (63.6)	21 (70.0)	10 (58.8)^d^	13 (92.9)^d^	0.045^d^	9.1^d^ (1.1–109.0)
Antibiotics	42 (48.5)	5 (37.5)	3 (60.0)	0.561		15 (46.9)	16 (53.3)	5 (29.4)	8 (57.1)	0.138	
NSAIDs	24 (28.2)	7 (50.0)	1 (25.0)	0.125		9 (29.0)	10 (33.3)	4 (23.5)	5 (35.7)	0.529	
Seizure Medication	0 (0.0)	0 (0.0)	1 (20.0)	0.054		0 (0.0)	0 (0.0)	0 (0.0)	1 (7.1)	0.298	
**Immunizations**											
Any Immunization	50 (57.5)^a^	10 (71.4)^b^	0 (0.0)^a,b^	0.017^a^	NC^a^	21 (63.6)	17 (56.7)	9 (52.9)	8 (57.1)	0.549	
				0.011^b^	NC^b^						
Influenza	37 (42.5)	7 (50.0)	0 (0.0)	0.080		15 (45.5)	14 (46.7)	5 (29.4)	6 (42.9)	0.356	
Hepatitis B	0 (0.0)^c^	2 (15.4)^c^	0 (0.0)	0.016^c^	NC^c^	2 (6.3)	0 (0.0)	0 (0.0)	0 (0.0)	0.492	
**Weight at Diagnosis**											
Overweight/Obese	23 (26.7)	1 (7.1)	1 (20.0)	0.117		10 (31.3)	7 (23.3)	2 (11.8)	3 (21.4)	0.174	
Underweight	6 (7.0)^c^	6 (42.9)^c^	0 (0.0)	0.002^c^	10.0^c^ (2.8–34.0)	3 (9.4)	4 (13.3)	1 (5.9)	3 (21.4)	0.304	
**Major Psychosocial Stressors**											
Overall Major Psychosocial Stress Score	0.0 [0.0–0.0]	0.0 [−0.3–0.0]	0.0 [0.0–0.0]	0.572		0.0 [0.0–0.0]	0.0 [0.0–0.0]	0.0 [0.0–0.0]	0.0 [0.0–0.0]	0.821	
Death or Illness of Significant Person	6 (6.9)	1 (7.1)	0 (0.0)	0.999		0 (0.0)	3 (10.0)	2 (11.8)	2 (14.3)	0.084	
**Exposures Beyond 1 Year of Diagnosis**											
**Major Psychosocial Stressors (within 5 Years of Diagnosis)**											
Overall Major Psychosocial Stress Score	0.0 [−1.0–0.0]	0.0 [−2.0–0.0]	0.0 [−1.0–0.0]	0.368		0.0 [−1.0–0.0]	0.0 [−2.0–0.0]	0.0 [−0.5–1.0]	0.0 [−1.3–0.0]	0.415	
Death or Illness of Significant Person	17 (19.5)	4 (28.6)	1 (20.0)	0.482		4 (12.1)^e^	9 (30.0)	2 (11.8)	6 (42.9)^e^	0.045^e^	5.4^e^ (1.3–19.6)
**Smoke Exposure**											
Maternal Smoking during Pregnancy	3 (5.2)	0 (0.0)	0 (0.0)	0.999		1 (4.3)	0 (0.0)	2 (18.2)	0 (0.0)	0.118	
Parental Smoking during Pregnancy	7 (13.2)	0 (0.0)	1 (50.0)	0.222		2 (10.0)	1 (5.3)	2 (18.2)	1 (16.7)	0.430	
Secondhand Smoke Exposure	7 (8.0)	3 (21.4)	2 (40.0)	0.073		3 (9.1)	2 (6.7)	2 (11.8)	2 (14.3)	0.581	
**Pregnancy Complications**											
C Section Birth	23 (36.5)	2 (28.6)	0 (0.0)	0.546		9 (37.5)	8 (40.0)	4 (33.3)	3 (33.3)	0.999	
Birth < 37 weeks	12 (19.3)	3 (42.9)	0 (0.0)	0.169		3 (12.5)	6 (31.6)	4 (33.3)	2 (22.2)	0.153	
Preeclampsia or Gestational Hypertension	4 (6.3)	0 (0.0)	0 (0.0)	0.999		0 (0.0)	3 (15.0)	1 (8.3)	1 (11.1)	0.086	
**Breastfed**											
Breastfed for any period	48 (78.7)	7 (100.0)	3 (100.0)	0.331		17 (70.8)	19 (95.0)^g^	7 (58.3)^g^	8 (88.9)	0.020^g^	13.6^g^ (1.7–166.1)
Breastfed for more than 6 months	25 (52.1)	5 (71.4)	1 (33.3)	0.443		7 (41.2)^e^	11 (57.9)	2 (28.6)^d^	8 (88.9)^d,e^	0.035^d^	20.0^d^ (1.5–256.5)
										0.036^e^	11.4^e^ (1.2–137.2)
Soy Formula	13 (21.0)	0 (0.0)	1 (33.3)	0.333		6 (26.1)	3 (15.0)	3 (27.3)	0 (0.0)	0.150	

*Abbreviations*: *JDM* juvenile dermatomyositis, *JPM* juvenile polymyositis, *JCTM* juvenile connective tissue myositis, *NSAIDs* non-steroidal anti-inflammatory drugs, *URI* upper respiratory Infection, *OR* odds ratio, *CI* confidence interval, *C section* caesarean section, *MSA* myositis-specific autoantibody

Note that percentages may not reflect the number divided by the total number of subjects when data are missing

Each individual comparison was computed, and the smallest *p* value is reported, except when more than one comparison is significant

ORs are reported for only significant results (*p* < 0.050) and are footnoted to delineate the specific comparison

Any infection, heavy exercise, medication, or vaccine refers to any exposure in the category, including those specifically listed in the table

^a-g^Denote the groups compared and the associated *p* value and OR

The frequency and number of sunburns within 12 months of diagnosis did not differ among clinical and serological subgroups, although all four anti-synthetase autoantibody positive patients reported at least one sunburn within 12 months of diagnosis.

The proportion of patients performing heavy exercise within 12 months of diagnosis differed among serologic phenotypes, with a greater proportion of MSA-negative patients reporting exercise resulting in muscle pain compared to patients with anti-TIF-1 autoantibodies (42.9% vs. 9.0%, *p* = 0.013). Further, a greater proportion of patients with anti-MDA5 autoantibodies reported a history of prolonged running within 12 months of diagnosis compared to patients with anti-TIF-1 autoantibodies (35.3% vs. 9.0%, *p* = 0.047).

MSA-negative patients more frequently received a medication within 12 months of diagnosis compared to patients with anti-MDA5 autoantibodies (92.9% vs 58.8%, *p* = 0.045), but specific types of medications did not differ among subgroups (Table 2). JDM and JCTM patients more frequently received an immunization within 12 months of diagnosis relative to patients with JPM (57.5 and 71.4% vs. 0.0%, *p* = 0.017 and 0.011 respectively). Among the specific immunizations administered, only JCTM patients received a hepatitis B vaccine within 12 months of diagnosis (15.4%, *p* = 0.016). No differences were observed among clinical and serologic subgroups in the frequency of other vaccines administered within 12 months of diagnosis, including influenza, tetanus, MMR, and polio vaccines.

A greater proportion of JCTM patients were underweight at diagnosis, defined by a BMI below the fifth percentile for age, relative to JDM patients (42.9% vs. 7.0%, *p* = 0.002). No differences were observed in the proportion of patients who were overweight or obese at diagnosis among JIIM subgroups.

While the frequency of major psychosocial stressors did not differ by clinical or serologic phenotype within 12 months of diagnosis, a greater proportion of patients who were MSA-negative reported experiencing the death or illness of a close individual (42.9%) within 5 years preceding diagnosis as compared to patients with anti-TIF-1 autoantibodies (12.1%, 0.045). However, major psychosocial stress impact scores did not differ among clinical or serologic subgroups at 1 or 5 years prior to diagnosis.

None of the JIIM patients reported a history of past or current smoking and there were no significant differences among the phenotypes in household exposures to secondhand smoke. No differences in prenatal smoke exposure from maternal, paternal, or combined parental sources were observed among clinical or autoantibody subgroups.

There were no differences in the frequency of birth complications among clinical and serologic groups, including delivery by Caesarean section, premature birth, preeclampsia or gestational hypertension, gestational diabetes, or maternal blood transfusion or receipt of Rhogam. A greater proportion of patients with anti-NXP2 autoantibodies were breastfed relative to patients with anti-MDA5 autoantibodies (95.0% vs. 58.3%, *p* = 0.020). Among those who were breastfed, a greater proportion of MSA-negative patients (88.9%) were breastfed for more than 6 months compared to those with anti-TIF-1 (41.2%, *p* = 0.036) and anti-MDA5 autoantibodies (28.6%, *p* = 0.035).

## Conclusions

Previous studies have identified environmental factors temporally correlated with the onset of different JIIM phenotypes, including prior associations with certain infections, UVR, as well as prenatal exposures to air pollution, maternal dust occupation, and tobacco smoke [6–11, 18–20]. Our study aimed to build upon these results by using refined questionnaires to examine a broader range of environmental exposures at a range of times prior to diagnosis in a well-characterized JIIM patient population.

A case-control study of adult dermatomyositis/polymyositis patients and their discordant siblings identified heavy exercise as a risk factor, which was similar to the findings observed in our study, with higher frequencies of certain heavy exercises among some serologic phenotypes [16]. The increased frequency of exercise in the anti-MDA5 autoantibody group was unexpected, given this group is characterized by mild muscle disease [21]. It is possible, however, that exercise-related exposures were painful or strenuous in part due to the presence of early myositis symptoms. The BEQ did not collect information regarding the duration and type of exercise leading to muscle pain. Further studies are required to determine whether this population has a lower exercise tolerance, particularly whether muscle dysfunction or muscle damage is contributing to initiation of disease.

We identified several new exposures seen more frequently prior to diagnosis in some clinical or serologic JIIM subgroups, which have been previously reported as risk factors for other autoimmune diseases or myositis. Gastroenteritis was more frequent in MSA negative patients, and gastrointestinal symptoms were previously reported in 30% of JDM patients preceding illness onset, with an increase in those with exposure to sick animals [9]. A dysbiososis of the gastrointestinal microbiome could be a potential factor in the initiation of these diseases, which has not been examined in JIIM. Our finding that certain psychosocial stressors differed among serologic phenotypes is consistent with prior studies that have reported psychosocial stress as a trigger for childhood autoimmune diseases and disease flare in JDM patients [22–24]. We found an increase in the frequency of certain immunizations, specifically the Hepatitis B vaccine, which differed among clinical subgroups. Previous studies have indicated a link between immunization and autoimmune-mediated events, although a study by Yu et al. found no increased risk of autoimmune thyroid disease following Hepatitis B vaccination [25–27]. A review on the associations between inflammatory myopathies and immunizations supports a potential causal link, including mechanisms of molecular mimicry, epitope spreading, reactivation of memory T cells, and release of autoantigens upon injection of foreign protein into the muscle, among others, although the occurrence appears to be rare and limited to genetically susceptible individuals [28].

Within our cohort, we observed an association of being underweight at diagnosis with JCTM. This is in contrast with previous studies of weight status and autoimmune diseases, which found obesity to be a risk factor for some autoimmune diseases [29, 30]. Several JCTM patients had overlapping conditions, such as celiac disease and scleroderma, that may be associated with malabsorption, although only one of these five patients with overlapping conditions was underweight at diagnosis.

Although we observed an increased frequency of being breastfed among certain serologic groups, the longer duration of breastfeeding in MSA-negative patients relative to other serologic groups may indicate a longer duration of breastfeeding is protective for severe illness. This is consistent with some studies of JIA, where a longer duration of being breastfed was found to be protective against the development of rheumatoid factor-positive polyarticular JIA [31]. Breastfeeding may provide a protective effect by eliminating early exposure to ingestion of complex proteins that may be antigenic, or alternatively, breast milk may provide autoantigens and the infant becomes immunologically desensitized [31].

We failed to observe previously reported clinical and serologic associations involving UV exposure, prenatal smoking, and infection, particularly respiratory infections [3, 6, 7, 11, 19]. Failure to observe a temporal association of sunburns prior to diagnosis of JDM and the myositis autoantibody phenotypes associated with JDM could be related to a high frequency of sunburns in healthy children [32]. For prenatal smoking, we failed to observe a higher frequency of maternal smoking among the subgroups, as was seen in Brazilian JDM patients, which may be related to a lower prevalence of maternal smoking during pregnancy in the United States [11, 33]. We did not confirm an association with respiratory infections prior to diagnosis in this cohort, in contrast to nationwide cohorts, which reported a high frequency of antecedent respiratory infections [7, 9]. The present study examined infections within 1 year of diagnosis, whereas the prior studies examined infections within a few months prior to JIIM symptom or illness onset.

There are a few potential limitations in this study. It is possible that the frequencies of certain exposures observed in juvenile myositis do not differ from those in a healthy population, as we did not have a control group of healthy children for comparison. Additionally, recall bias is inherent with the study design, resulting in the potential for over- or under-reporting of exposures. By focusing on diagnosis date, as opposed to start of JIIM symptoms, there may be under-reporting of certain exposures, although symptom onset date is often not a reliable timepoint. Also, this study is underpowered for certain subgroups resulting in missed associations and risk factor estimates with wide confidence intervals. Certain exposures, such as birth order, were not examined, and others were not deeply probed in this exploratory study. Adjustment for confounding factors, such as age, disease duration and socioeconomic status, is also needed in future larger studies. These limitations should be addressed through the completion of large, prospective cohort studies with well-characterized subgroups, in addition to case-control studies, to confirm that these exposures are truly associated with specific phenotypes of JIIM. For some exposures, we are collecting additional data as part of other studies on environmental factors to examine in more depth.

In summary, we have identified potential environmental exposures prior to diagnosis that may differ among clinical and serologic subgroups and may be important in the development of JIIM phenotypes. Specifically, we observed certain immunizations, a psychosocial stressor, and heavy exercise prior to diagnosis, as well as prolonged breastfeeding to be increased in specific JIIM phenotypes, which should be explored as possible new environmental risk factors. These distinct exposures among subgroups may be useful in understanding the pathogenesis of JIIM. However, the findings observed in this small, retrospective study require confirmation in prospective studies with larger numbers of patients in each subgroup.

## Supplementary Information

**Additional file 1: Supplemental Table 1**. Environmental Questionnaire Content.

## Data Availability

The datasets generated and analyzed during the current study are not publicly available due to concerns regarding patient privacy, but are available from the corresponding author on reasonable request.
