# Supplementary material for: Environmental factors associated with juvenile idiopathic inflammatory myopathy clinical and serologic phenotypes
Source: Pediatr Rheumatol Online J. 2022 Apr 12;20:28. doi: 10.1186/s12969-022-00684-9 (PMC9004071; doi:10.1186/s12969-022-00684-9)
Supplement: Supplementary file 1 — Additional file 1: Supplemental Table 1. Environmental Questionnaire Content. [file 12969_2022_684_MOESM1_ESM.docx]

| **Exposure** | **Questionnaire** | **Questions** |
| --- | --- | --- |
| Immunizations | Brief Environmental Questionnaire | •Any immunization within 12 months of diagnosis  •Type of vaccination: specific vaccines queried were influenza, swine flu, hepatitis B, tetanus, MMR, mumps, whooping cough, rubella, varicella, diphtheria, DT, polio, and tuberculosis |
| Medication use | Brief Environmental Questionnaire | •Usage of any medication within 12 months of diagnosis  •Type of medication: specific medications queried were cholesterol-lowering drugs, antibiotics, NSAIDs, blood pressure medication, medicine for depression, medicine for diabetes (except insulin), seizure medication, acne medication |
| Infections | Brief Environmental Questionnaire | •Diagnosis of an infection requiring prescription medication within 12 months of diagnosis  •Type of infection: specific infections included skin infection, influenza, UTI, strep throat (treated with antibiotics), pneumonia, hepatitis, stomach virus or gastroenteritis, genital infection, sinus infection (treated with antibiotics), tooth or gum infection, URI |
| UV Exposure | Brief Environmental Questionnaire | •Number of sunburns within 12 months prior to diagnosis  •Frequency of sunbathing  •Skin type (very fair, fair, olive, light brown, dark brown, very dark)  •Degree of burning and tanning (always burns/never tans, always burns easily/tans minimally, burns moderately/tans uniformly, burns minimally/always tans well, rarely burns/tans profusely, never burns |
| Exercise Status | Brief Environmental Questionnaire | •Engagement in heavy exercises 12 months prior to diagnosis including weightlifting, prolonged running, strenuous sports activity, and exercise resulting in muscle pain  •Average activity level 12 months and 5 years prior to diagnosis (sedentary, moderate, moderate to regular, and regular levels of activity) |
| Smoking | Brief Environmental Questionnaire | •100 lifetime cigarettes- ever smoking, current smoking  •Packs of cigarettes smoked per day on average  •Living, working, or going to school where someone regularly smokes indoors, and years exposed |
| Major Psychosocial Stressors | Brief Environmental Questionnaire | •Occurrence of major stressful life events within 5 years and 1 year of diagnosis   - Major stressful life events probed were moving to another house, death or illness of person close to participant, divorce or separation of parents, start of marriage of parents, change of schools, increased level or decreased level of responsibility at school, start of unemployment   •Impact of event (very negative, negative, neutral, positive, very positive) |
| Prenatal Smoke Exposure | Pollution and Prenatal Questionnaire or Twin/Sibling Study Questionnaire | •Smoking of mother while pregnant with participant  •Smoking of father while mother was pregnant with participant |
| Pregnancy Complications | Pollution and Prenatal Questionnaire or Twin/Sibling Study Questionnaire | •Occurrence of birth/prenatal complications: specific birth/prenatal complications queried included maternal history of gestational diabetes, gestational hypertension, pre-eclampsia, Caesarian section delivery, blood transfusion or Rhogam, fever, placenta previa; other pregnancy complications |
| Breastfeeding | Pollution and Prenatal Questionnaire or Twin/Sibling Study Questionnaire | •Occurrence and duration of breastfeeding  •Use of soy formula |

Abbreviations: MMR, measles, mumps, rubella; DT, diphtheria and tetanus; NSAIDs, non-steroidal anti-inflammatory drugs; UTI, urinary tract infection; URI, upper respiratory infection; C section, caesarean section

*The Brief Environmental Questionnaire also included questions on occupational and hobby exposures, though these were excluded from the analysis due to low frequency rates among juvenile-onset patients
